# Clinical significance of splenic switch-off in adenosine triphosphate ^13^N-ammonia positron emission tomography in patients without coronary artery disease

**DOI:** 10.1007/s11604-025-01762-0

**Published:** 2025-03-29

**Authors:** Kentaro Ohara, Naoto Kawaguchi, Hideki Okayama, Hitomi Ueda, Ryutaro Ueno, Kuniaki Hirai, Yuki Tanabe, Megumi Matsuda, Tomoyuki Kido, Takeshi Inoue, Teruhito Kido

**Affiliations:** 1 Department of Radiology, Ehime Prefectural Central Hospital, Matsuyama, Ehime Japan; 2 Department of Radiology, Ehime University Graduate School of Medicine, Toon, Ehime 791-0295 Japan; 3 Department of Cardiology, Ehime Prefectural Central Hospital, Matsuyama, Ehime Japan

**Keywords:** ^13^N-ammonia PET, Splenic switch-off, Myocardial flow reserve, Coronary microvascular dysfunction

## Abstract

**Purpose:**

Splenic switch-off (SSO) is defined as a decrease in splenic radiotracer uptake following pharmacological stress. This study aimed to assess the clinical utility of SSO on adenosine triphosphate (ATP) ^13^N-ammonia positron emission tomography (PET) in patients without coronary artery disease (CAD).

**Materials and Methods:**

We analyzed 63 patients (mean age, 67 ± 11 years; 34 males) who underwent ATP ^13^N-ammonia PET without significant CAD on invasive coronary angiography or cardiac computed tomography within 6 months. Visual assessment of the SSO was conducted by two independent observers, with disagreements resolved by a third observer. We divided the patients into two groups according to the presence (positive) or absence (negative) of SSO and compared global myocardial flow reserve (MFR) and myocardial blood flow (MBF) between the two groups. In addition, the relationship between the reduced MFR and SSO was investigated.

**Results:**

Negative SSO was observed in 15 of 63 patients. Global MFR and global stress MBF were significantly higher in the positive SSO group than in the negative SSO group (2.4 ± 0.6 vs. 1.6 ± 0.6, *P* < 0.001, and 2.3 ± 0.5 vs. 1.5 ± 0.5 ml min^−1^ g^−1^, *P* < 0.001, respectively). Global rest MBF showed no significant difference between the two groups (1.0 ± 0.2 vs. 1.0 ± 0.3 ml min^−1^ g^−1^, *P* = 0.80). In the negative SSO group, 12 of the 15 (80%) patients had a reduced MFR (<2.0). In contrast, in the positive SSO group, 15 of the 48 (31.3%) patients had a reduced MFR, which was suggestive of coronary microvascular dysfunction (CMD).

**Conclusions:**

The presence of a splenic response based on the visual assessment of SSO may be used to identify an adequate pharmacological response. This can affect the diagnosis of CMD in patients with reduced MFR without CAD.

**Secondary abstract:**

This study showed the clinical utility of splenic switch-off in adenosine triphosphate ^13^N-ammonia positron emission tomography in patients without coronary artery disease.

**Supplementary Information:**

The online version contains supplementary material available at 10.1007/s11604-025-01762-0.

## Introduction

Positron emission tomography (PET) myocardial perfusion imaging (MPI) is a powerful tool for the diagnosis of myocardial ischemia, allowing qualitative and quantitative assessment of MPI [1–5]. In PET-MPI, both resting and pharmacological stress images are acquired to calculate the myocardial flow reserve (MFR) used for quantitative assessment. However, their accuracy depends on whether an adequate response to pharmacological stress has been achieved.

Splenic switch-off (SSO) has been used as a marker of an adequate response to pharmacological stress [1, 6–8]. SSO describes a decrease in the splenic signal intensity or uptake of splenic radiotracers after vasodilatory stress [1]. This is presumably due to reduced splenic blood flow, which may be mediated by reactive sympathetic vasoconstriction after pharmacologically induced hypotension [7, 9].

Previously, SSO was used in cardiac magnetic resonance [10–12], but recently, its usefulness has also been reported in PET [1, 6–8]. A recent study showed that the absence of SSO could be used to identify patients who are inadequately stressed by ^82^Rb-PET [1].

However, previous studies have focused on patients with coronary artery disease (CAD), and insufficient studies have been conducted on patients without CAD. Hence, it is unclear whether the decrease in MFR is due to the absence of SSO or CAD.

We thought that examining the relationship between the presence or absence of SSO and MFR in cases without CAD would clarify whether the decrease in MFR is due to the absence of SSO and that SSO can be used as an appropriate pharmacological marker. In addition, the presence or absence of SSO, which can be easily assessed, may contribute to the diagnosis of CAD.

This study aimed to assess the relationship between MFR and the presence or absence of SSO in patients without CAD and to clarify whether SSO can be used as an appropriate pharmacological marker.

## Methods

### Study design and population

We surveyed 791 consecutive patients who underwent adenosine triphosphate (ATP) stress ^13^N-ammonia PET between April 2014 and March 2024. Among them, 457 patients who underwent invasive coronary angiography (ICA) or cardiac computed tomography (CT) within 6 months of PET were selected. The exclusion criteria were as follows: (1) significant stenosis (>50%) in ICA or cardiac CT, (2) previous percutaneous coronary intervention (PCI) and coronary artery bypass grafting (CABG), (3) unstable angina, (4) congenital heart disease, (5) greater than first-degree atrioventricular block, (6) symptomatic asthma, and (6) poor image quality.

We excluded 394 patients based on the following exclusion criteria (significant stenosis, n = 355; PCI/CABG, n = 34; congenital heart disease, n = 3; poor image quality, n = 2). Finally, 63 patients were included in the final analysis.

The institutional ethics committee approved this retrospective study protocol (Ehime Prefectural Central Hospital, No. 05–30), which waived the need for informed consent because of the retrospective observational nature of this study. The opt-out method was used to obtain consent for this study.

### Positron emission tomography (PET) acquisition protocol

All patients underwent single-day rest/stress ^13^N-ammonia PET using a PET/CT scanner (Discovery STE, GE Healthcare, Milwaukee, USA before 2019; Discovery IQ.x, GE Healthcare, Milwaukee, USA after 2019). Dynamic PET scans at rest and during pharmacological stress were performed in list mode, and dynamic frames were reconstructed (12 × 10 s, 4 × 60 s, 2 × 120 s, and 1 × 10 min, for a total of 20 min) [13].

CT (120 kVp) was used for attenuation correction. The patients were instructed to fast for >6 h and were instructed to refrain from consuming beverages containing caffeine for at least 12 h before the test. Current smokers were instructed to abstain from smoking for at least 24 h before the test to eliminate the acute effects of smoking [14, 15]. Additionally, patients were advised to avoid using coronary dilators for at least 24 h before the test. The rest of the scan was performed using 370 MBq ^13^N-ammonia as a bolus injection with a saline flush. After 1 h, the pharmacological stress scan was performed during ATP-induced hyperemia at a rate of 160 μg kg^–1^ min^–1^ over 5–6 min. After a 4-min ATP infusion, ^13^N-ammonia was injected at the same dose, and the same protocol described above was followed. The heart rate (HR) was recorded at the time of the rest test and every minute during and after ATP infusion, with continuous electrocardiography (ECG) monitoring. Blood pressure (BP) was simultaneously measured at the ankle using a sphygmomanometer.

### Quantitative PET analysis

PET images were quantitatively analyzed using a commercially available software package (Syngo MBF; Siemens Healthineers, Forchheim, Germany). Quantitative analysis was almost automatic using a two-compartment model for the model previously developed by Hutchins et al. [16]. The global MFR was calculated as the ratio of the global stress myocardial blood flow (MBF) to the remaining MBF. Reduced MFR was defined as ≤2.0 [17].

### Visual PET analysis

Visual PET analysis was performed by N.K., a specialist in cardiovascular nuclear imaging with >10 years of experience. The presence or absence of fill-in was evaluated by visually comparing the qualitative polar maps of the stress and rest PET perfusion images. Visually present fill-in was defined as an improvement in the remaining polar map of the hypoperfusion area on the stress polar map.

### Visual assessment of splenic switch-off (SSO)

**Fig. 1:**
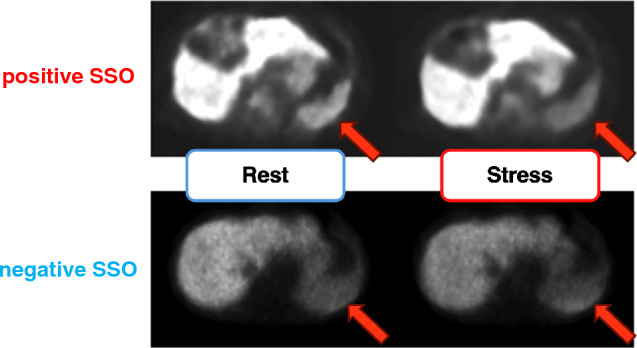
Visual assessment of SSO. Visual SSO was evaluated by visually comparing the uptake of radioactive tracers in the spleen as observed in the PET images. A positive SSO was defined as a clear decrease in splenic radiotracer uptake from rest to stress. Negative SSO was defined as a visually similar splenic radiotracer uptake at rest and during stress. *SSO* splenic switch-off, *PET* positron emission tomography

Visual assessment of the SSO was conducted by two independent observers, K.O. and K.H. Disagreements were resolved by a third observer, N.K. Visual SSO was evaluated by visually comparing the uptake of radioactive tracers in the spleen as observed in the PET images (Fig. 1). Visually present SSO was defined as a clear decrease in the splenic radiotracer uptake from rest to stress. An absent visual SSO was defined as a visually similar splenic radiotracer uptake at rest and during stress [1]. The study population was stratified into two groups based on the presence or absence of SSO.

### Quantitative assessment of splenic switch-off (SSO)

The standardized uptake value (SUV) (Bq g^−1^) of the spleen, liver, and myocardium was measured under resting and stress conditions by two investigators (K.O. and K.H.), who were blinded to each other’s results. Spherical volumes of interest (VOIs) between 10 and 20 mm were manually measured twice, and the average values of SUV mean for each region were obtained [1, 18]. VOIs were placed in the center of the liver or spleen, avoiding proximity to the organ’s borders and splenic regions with overlapping renal radiotracer uptake or liver VOIs containing large blood vessels. Myocardial VOIs were placed along the left ventricle wall, avoiding its edges.

A random subset of 10 patients was used to measure inter- and intra-rater reproducibility. For intra-observer reliability, the same investigator (K.O.) reassessed the SUVs in this subset. For inter-observer reliability, the second investigator (K.H.) evaluated SUVs on the same subset following the same protocol as previously described.

### Calculation of splenic response ratio (SRR)

The liver was used as a control organ to normalize the splenic radiotracers concentrations as in previous studies [1, 18]. The spleen to liver ratio during rest (SLRrest) was defined as SLRrest = splenic SUVmean rest/liver SUVmean rest, and the spleen to liver ratio during stress (SLRstress) was defined as SLRstress = splenic SUVmean stress/liver SUVmean stress. SRR was calculated as SRR = SLRstress/SLRrest. Patients with an SRR ≤ 0.88 were classified as splenic responders, while patients above that were classified as non-responders, as described by Saad et al. [1].

### Statistical analyses

Normality of the data was assessed using the Shapiro–Wilk test. To compare the data between the two groups, Student’s t-tests were used for normally distributed data, Mann–Whitney U tests were used for non-normally distributed data, and chi-squared tests were used for categorical data. Multiple logistic regression analysis was performed to explore the factors that affect the reduced MFR. Intra- and inter-observer reliability for SUV measurement was assessed by the intraclass correlation coefficient (ICC). To compare the changes in SUV from rest to stress, paired t-tests were used for normally distributed data, and Wilcoxon signed-rank sum test were used for non-normally distributed data. Statistical analyses were performed using the R statistical software (version 4.2.2) [19]. *P* < 0.05 was considered statistically significant.

## Results

Sixty-three patients were included in our study. Based on the visual assessment, 48 and 15 patients were in the positive and negative SSO groups, respectively. The baseline characteristics are presented in Table 1. Sex and smoking status significantly differed between the two groups (*P* < 0.05).

**Table 1:** Baseline characteristics

	Negative SSO (n = 15)	Positive SSO (n = 48)	*P*-value
Age (years)	65.6 ± 12.5	67.4 ± 10.1	0.58
Male sex (n, %)	12 (80%)	22 (46%)	0.036
BMI (kg/m^2^)	23.7 ± 3.3	23.8 ± 3.6	0.87
Risk factor (n, %)			
Hypertension	11 (73%)	35 (73%)	1
Diabetes mellitus	6 (40%)	21 (44%)	1
Dyslipidemia	6 (40%)	34 (71%)	0.06
Family history of heart disease	5 (33%)	19 (35%)	0.77
Smoking (n, %)	12 (80%)	21 (44%)	0.019
Never	3 (20%)	27 (56%)	
Past	5 (33%)	18 (38%)	
Current	7 (47%)	3 (6%)	
CKD (eGFR ≤ 30) (n, %)	5 (33%)	3 (6%)	0.015
Chest pain (n, %)	8 (53%)	34 (71%)	0.23
Mediation (n, %)			
Beta-blocker	4 (27%)	9 (19%)	0.49
Calcium channel blocker	4 (27%)	22 (46%)	0.24
RAS inhibitor	4 (27%)	12 (25%)	1
Diuretic	1 (7%)	3 (6%)	1
Coronary dilator	3 (20%)	8 (17%)	0.71

Data given as means ± SDs or numbers (%)

*SSO* splenic switch-off, *BMI* body mass index, *CKD* chronic kidney disease, *eGFR* estimated glomerular filtration rate, *RAS* renin–angiotensin system

Factors contributing to the decreased MFR were analyzed using univariate and multivariate analyses. (Table 2). Consequently, the model chi-square test result was significant (*P* = 0.03), identifying SSO as the only independent factor for decreased MFR (odds ratio (OR) = 9.36, 95% confidence interval (CI): 1.93–45.4, *P* = 0.006).

**Table 2:** Univariate and multivariate analysis to identify clinical variables that differentiate reduced global MFR

	MFR < 2.0 (n = 27)	MFR ≥ 2.0 (n = 36)	*P*-value (univariate)	*P*-value (95% CI) (multivariate)
Age (years)	68.0 ± 12.1	66.2 ± 9.8	0.51	0.40 (0.92–1.03)
Male sex (n, %)	16 (59%)	18 (50%)	0.61	0.97 (0.21–4.57)
BMI (kg/m^2^)	23.8 ± 3.9	23.8 ± 3.2	0.99	0.68 (0.82–1.14)
Risk factor (n, %)				
Hypertension	21 (78%)	25 (69%)	0.57	
Diabetes mellitus	13 (48%)	14 (39%)	0.61	
Dyslipidemia	17 (63%)	23 (64%)	1	
Family history of heart disease	12 (44%)	12 (33%)	0.44	
Smoking (n, %)	15 (56%)	18 (50%)	0.8	0.70 (0.28–6.62)
CKD (eGFR ≤ 30) (n, %)	6 (22%)	2 (6%)	0.06	0.42 (0.07–3.09)
Chest pain (n, %)	16 (59%)	26 (72%)	0.30	
Visual SSO	15 (56%)	33 (92%)	0.002	0.006 (1.93–45.4)

*MFR* myocardial flow reserve, *BMI* body mass index, *CKD* chronic kidney disease, *SSO* splenic switch-off

Hemodynamic responses to pharmacological stress are shown in Table 3. HR measurements were obtained for all patients, whereas BP measurements could not be completed for four patients, with three patients missing data during rest and four patients missing data during stress testing. The HR change rate and the number of patients with a significantly increased HR (≥10/min) were greater in the positive SSO group than in the negative SSO group, but the difference was not significant. Similarly, the systolic blood pressure (SBP) and the diastolic blood pressure (DBP) change rate showed no significant difference between the two groups. We also stratified global MFR by SSO and HR (Supplementary Table 1). No significant differences in MFR were observed between positive and negative SSO groups when classified by increased HR.

**Table 3:** Hemodynamic response

	Negative SSO (n = 15)	Positive SSO (n = 48)	*P*-value
HR change rate (%)	18.8 ± 21.0	25.3 ± 16.2	0.21
Pre-stress HR (bpm)	66.9 ± 12.6	64.7 ± 9.7	0.48
Post-stress HR (bpm)	78.6 ± 15.0	80.6 ± 13.1	0.63
Significantly increased HR (≥ 10) (n, %)	7 (47%)	36 (75%)	0.06

	Negative SSO (n = 11)	Positive SSO (n = 47)	*P*-value
SBP change rate (%)	1.2 ± 46.0	−14.7 ± 17.6	0.06
Pre-stress SBP (mmHg)	146.9 ± 36.7	153.5 ± 25.0	0.48
Post-stress SBP (mmHg)	140.6 ± 21.6	129.5 ± 28.1	0.23
DBP change rate (%)	−7.8 ± 15.0	− 2.8 ± 28.1	0.56
Pre-stress DBP (mmHg)	84.3 ± 15.2	80.0 ± 15.0	0.40
Post-stress DBP (mmHg)	77.0 ± 19.6	75.3 ± 15.7	0.75

Data given as means ± SDs or numbers (%)

Four patients from the group without SSO and one patient from the group with SSO were excluded from the analysis due to missing BP

*HR* heart rate, *BP* blood pressure, *SBP* systolic blood pressure, *DBP* diastolic blood pressure, *SSO* splenic switch-off

HR change rate = (post-stress HR − pre-stress HR)/pre-stress HR × 100

SBP change rate = (post-stress SBP − pre-stress SBP)/pre-stress SBP × 100

DBP change rate = (post-stress DBP − pre-stress DBP)/pre-stress DBP × 100

### Quantitative PET parameters between the positive and negative SSO groups

**Fig. 2:**
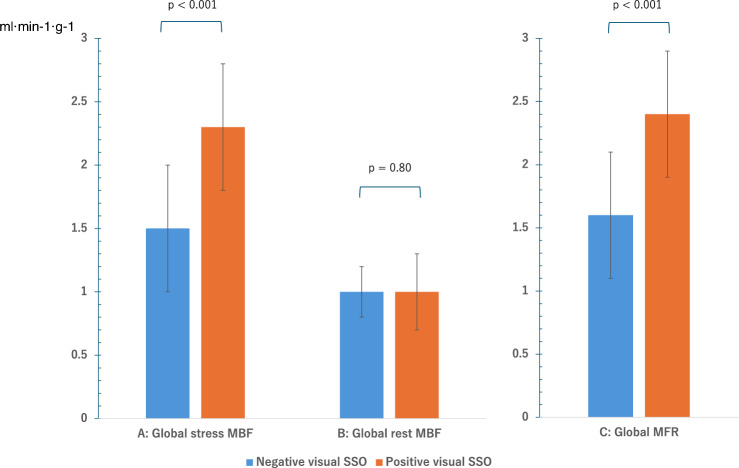
Results of quantitative PET parameters. Global stress MBF (**A**) and global MFR (**C**) were significantly higher in the SSO group than in the absent SSO group (mean, 2.3 ± 0.5 vs. 1.5 ± 0.5 ml min^−1^ g^−1^ and 2.4 ± 0.6 vs. 1.6 ± 0.6, respectively). Global rest MBF (**B**) showed no significant difference between the two groups. *PET* positron emission tomography, *SSO* splenic switch-off, *MBF* myocardial blood flow, *MFR* myocardial flow reserve

Figure 2 shows the quantitative PET parameters. Global stress MBF was significantly higher in the positive SSO group (2.3 ± 0.5 ml min^−1^ g^−1^) compared with the negative SSO group (1.5 ± 0.5 ml min^−1^ g^−1^) (*P* < 0.001). Similarly, global MFR was significantly higher in the positive SSO group (2.4 ± 0.6) than in the negative SSO group (1.5 ± 0.6) (*P* < 0.001). There was no significant difference in global rest MBF between the two groups (1.0 ± 0.2 vs. 1.0 ± 0.3 ml min^−1^ g^−1^, *P* = 0.81).

We also investigated the corrected rest MBF and corrected MFR using the rate pressure product (RPP) (Supplementary Table 2). Even after correction using RPP, the global rest MBF and MFR values were almost identical.

### Relationship between reduced myocardial flow reserve and SSO

**Fig. 3:**
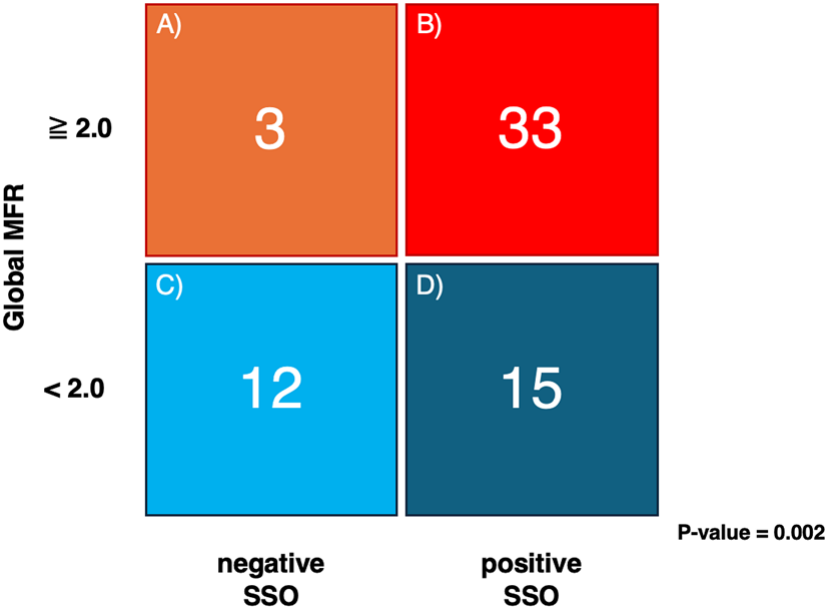
2 × 2 contingency table analyzing the relationship between global MFR and SSO. The global MFR was 2.0, and the SSO was divided into positive and negative values. Group **A**: 3 patients with global MFR ≥ 2.0 and negative SSO. Group **B**: 33 patients with global MFR ≥ 2.0 and positive SSO. Group **C**: 12 patients with global MFR < 2.0 and negative SSO. Group **D**: 15 patients with global MFR < 2.0 and positive SSO. *MFR* myocardial flow reserve, *SSO* splenic switch-off

The study cohort was stratified into four groups based on the global MFR and SSO (Fig. 3). The groups were defined as follows: (A) global MFR ≥ 2 without SSO, (B) global MFR ≥ 2 with SSO, (C) global MFR < 2 without SSO, and (D) global MFR < 2 with SSO. There were 3, 33, 12, and 15 patients in groups A, B, C, and D, respectively. In the negative SSO group, 80.0% of the patients had a reduced MFR (< 2.0), whereas in the positive SSO group, only 31.3% of the patients had a reduced MFR (*P* < 0.001).

**Fig. 4:**
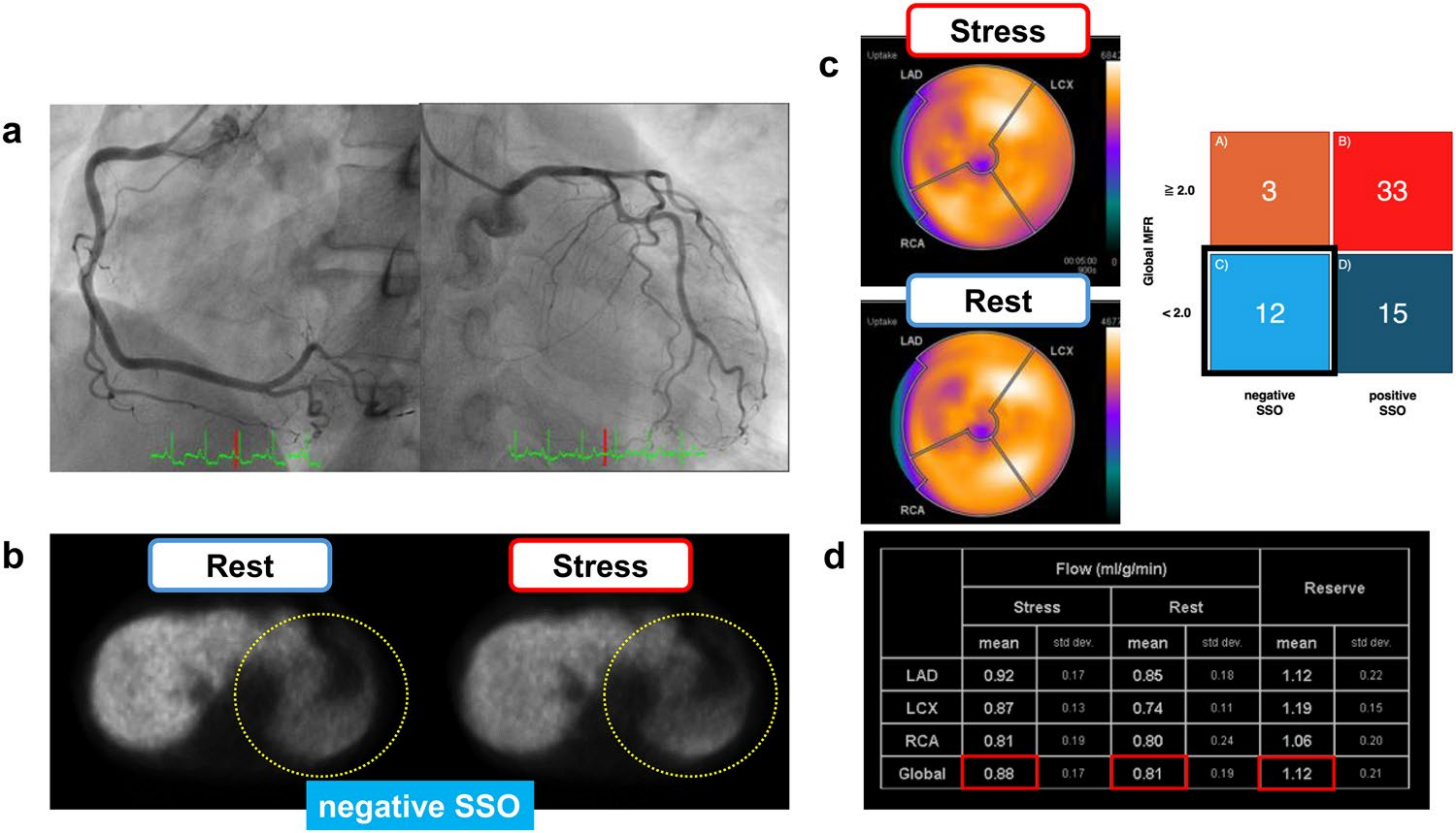
Representative case of group C. Male patient in his 60 s with dyslipidemia and a current smoker presented with chest pain. **a** Invasive coronary angiography showed no significant coronary stenosis. **b** The SSO was judged as negative, suggesting an inadequate response to pharmacological stress. **c** Qualitative assessment of myocardial perfusion showing no fill-in patterns. **d** In the quantitative assessment, global stress MBF and global MFR were <2.0 (*red square line*). This patient had a diffusely low MFR, no significant coronary stenosis, negative SSO, and a poor heart rate increase during ATP infusion, suggesting an inadequate response to pharmacological stress. *SSO* splenic switch-off, *MBF* myocardial blood flow, *MFR* myocardial flow reserve, *LAD* left anterior descending artery, *LCX* left circumflex artery, *RCA* right coronary artery, *ATP* adenosine triphosphate

**Fig. 5:**
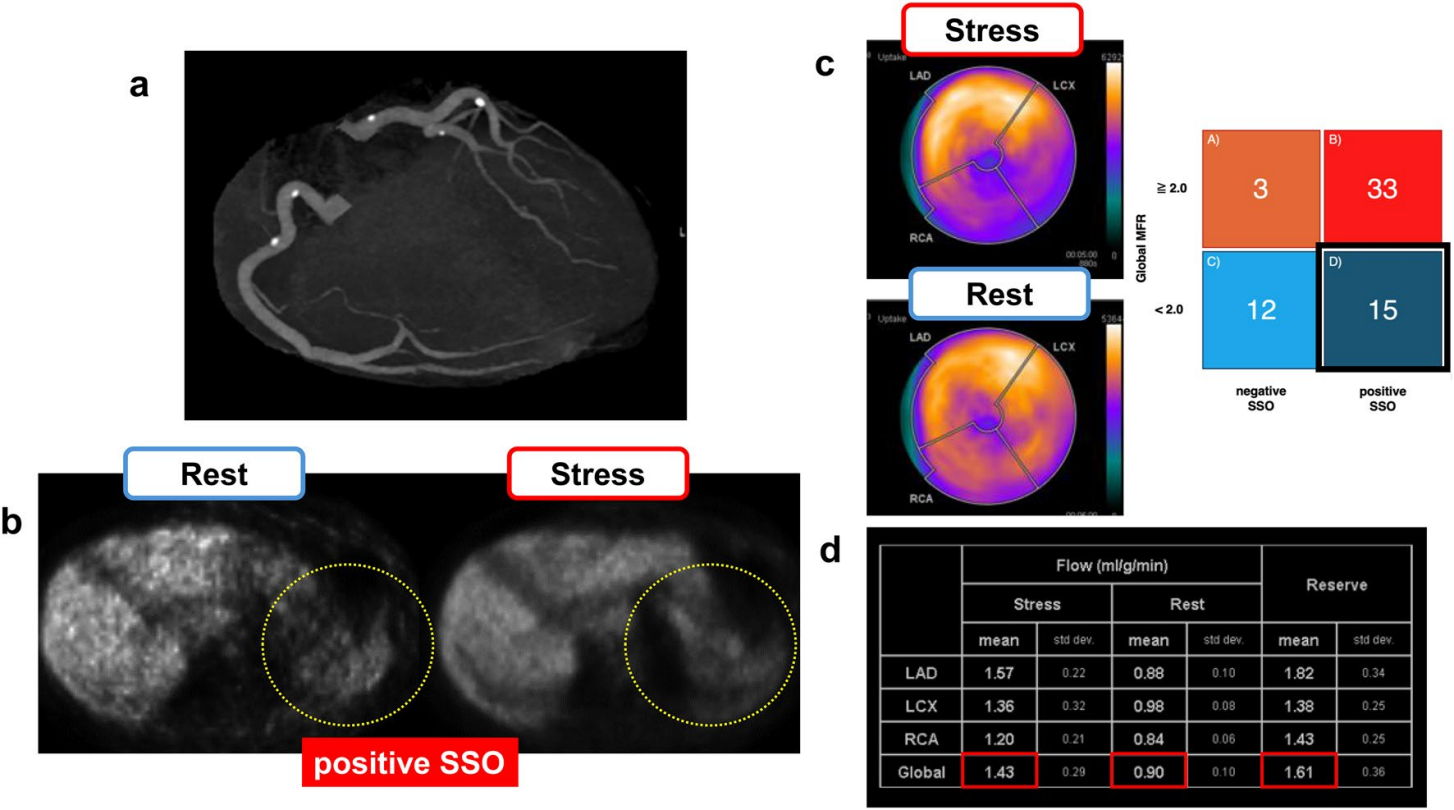
Representative case of group D. Male in his 50s with hypertension, diabetes mellitus, and dyslipidemia and a current smoker presented with chest pain. **a** Cardiac CT showed no significant stenosis. **b** SSO was judged positive, suggesting an adequate response to pharmacological stress. **c** Qualitative assessment of myocardial perfusion showing a fill-in pattern in the LCX and RCA. **d** In the quantitative assessment, global stress MBF and global MFR were <2.0. As in the qualitative assessment, the MFR in the RCA and LCX was markedly reduced (*red square line*). The patient had no significant coronary stenosis or typical chest pain, and INOCA was suspected. PET showed ischemia (fill-in pattern) and decreased MFR, suggesting a high-risk group for MACE. *SSO* splenic switch-off, *MBF* myocardial blood flow, *MFR* myocardial flow reserve, *LAD* left anterior descending artery, *LCX* left circumflex artery, *RCA* right coronary artery, *INOCA* ischemic nonobstructive coronary artery disease, *MACE* major adverse cardiovascular events

In this study, which excluded patients with CAD, the cause of reduced MFR may have been coronary microvascular dysfunction (CMD) or inadequate pharmacological stress. Group D, with a positive SSO, suggests that CMD is likely to cause a reduced MFR. In group C, with negative SSO, not only CMD but also inadequate pharmacological stress should be considered a cause of reduced MFR. Figures 4 and 5 show representative cases from groups C and D, respectively.

### Quantitative evaluation of splenic response

Two patients were excluded from the analysis because the SUV could not be measured. The SUVmean changes from rest to stress for each organ are shown in Table 4. The spleen SUVmean significantly decreased from rest to stress (3.5 ± 0.9 vs. 2.5 ± 0.8 Bq g^−1^, respectively, *P* < 0.001). There was a slight but significant decrease in the liver SUVmean (7.0 ± 1.4 vs. 6.7 ± 1.9 Bq g^−1^, respectively, *P* = 0.049). The myocardium SUVmean significantly decreased from rest to stress (9.3 ± 2.1 vs. 11.8 ± 4.2 Bq g^−1^, respectively, *P* < 0.001). There was high intra- and inter-rater reliability with measurements of spleen, liver, and myocardium SUVmean (Intra-class correlation, ICC > 0.90).

**Table 4:** SUVmean changes from rest to stress

	Rest	Stress	*P*-value
Spleen SUVmean changes from rest to stress (Bq g^−1^)	3.5 ± 0.9	2.5 ± 0.8	<0.001
Liver SUVmean changes from rest to stress (Bq g^−1^)	7.0 ± 1.4	6.7 ± 1.9	0.049
Myocardium SUVmean changes from rest to stress (Bq g^−1^)	9.3 ± 2.2	11.8 ± 4.2	<0.001

Data given as means ± SDs or numbers (%)

*SUVmean* standardized uptake value mean

**Fig. 6:**
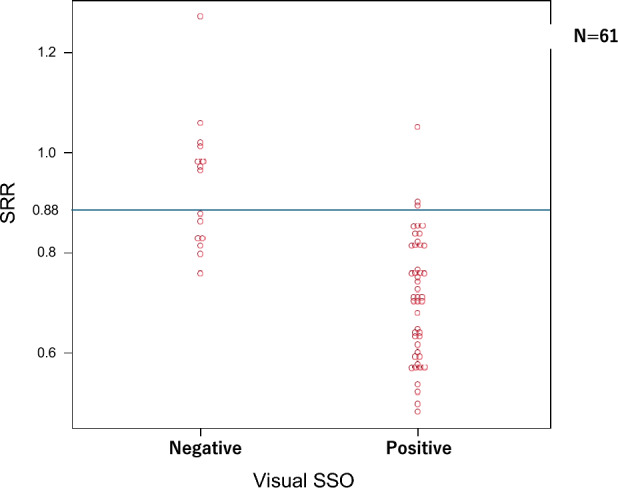
The relationship between visual SSO and SRR. The SRR of positive SSO was significantly lower than that of negative SSO (mean, 0.70 ± 0.12 vs. 0.93 ± 0.13, respectively, *P* < 0.001). The cutoff line for SRR is shown at 0.88. *SSO* splenic switch-off, *SRR* splenic response ratio

Of the patients, 50 (82%) were classified as splenic responders (SRR ≤ 0.88), and 11 (18%) as non-responders (SRR > 0.88). Figure 6 shows the relationship between visual SSO and SRR. The SRR of positive SSO was significantly lower than that of negative SSO (0.70 ± 0.12 vs. 0.93 ± 0.13, respectively, *P* < 0.001). Using a cutoff of 0.88, SRR showed discordance with seven cases of negative SSO as responders and three cases of positive SSO as non-responders. Supplementary Fig. 1 shows the quantitative PET parameters. Global stress MBF was significantly higher in the responder group (2.2 ± 0.5 ml min^−1^ g^−1^) compared with the non-responder group (1.5 ± 0.6 ml min^−1^ g^−1^) (*P* < 0.001). Similarly, global MFR was significantly higher in the responder group (2.3 ± 0.7) than in the non-responder group (1.6 ± 0.7) (*P* = 0.004).

The study cohort was also stratified into four groups based on the global MFR and SSR as the relationship between reduced MFR and the responders (Supplementary Fig. 2).

### Clinical decision pathway for ischemia without obstructive arteries using ^13^N-ammonia PET-myocardial perfusion imaging including SSO

**Fig. 7:**
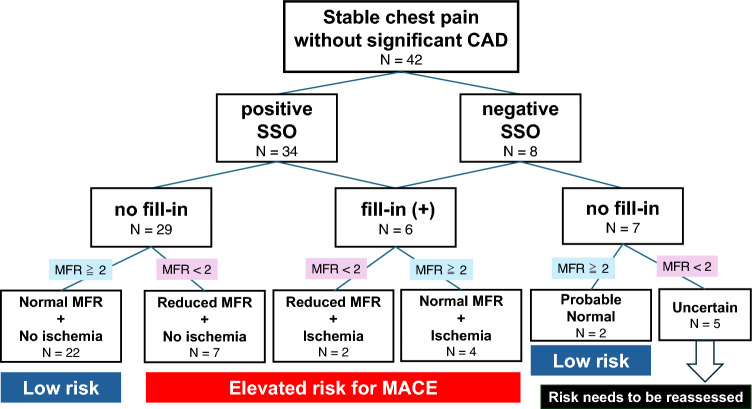
The flowchart of clinical decision pathway for INOCA using ^13^N-ammonia PET-MPI including SSO. This figure presents a flowchart analyzing the risk stratification in 42 patients with stable chest pain without significant CAD, divided based on the presence of SSO and MFR values. This flowchart aids in assessing the risk of MACE based on a combination of SSO and MFR values. *INOCA* ischemic nonobstructive coronary artery disease, *CAD* coronary artery disease, *SSO* splenic switch-off, *MFR* myocardial flow reserve, *MACE* major adverse cardiovascular events

In the present study, 42 (67%) patients had chest pain suggestive of ischemia with non-obstructive coronary arteries (INOCA). Figure 7 shows a flowchart of the clinical decision pathway for INOCA using PET, including SSO, based on the Guideline for the Evaluation and Diagnosis of Chest Pain [20]. Of the 42 patients, 34 and 8 were in the positive and negative SSO groups, respectively. In the positive SSO group, those without fill-in and reduced MFR had a low risk of major adverse cardiovascular events (MACE), whereas the presence of either increased the risk of MACE. In the negative SSO group, when the patient with fill-in was considered to have adequate pharmacological stress and was judged equivalent to the positive SSO group, when the MFR was normal with no fill-in, the patient was probably normal and was diagnosed at low risk; when both no fill-in and reduced MFR were present, the patient might have inadequate pharmacological stress and risk should be reassessed.

## Discussion

The main findings of this study were as follows: (1) both global stress MBF and MFR were significantly higher in the positive SSO group than in the negative SSO group and (2) in the negative SSO group, 12 of 15 (80%) patients had a reduced MFR (<2.0). In contrast, in the positive SSO group, 15 of 48 (31.3%) patients had a reduced MFR, suggestive of CMD.

In the present study, we showed that the presence of SSO may be a marker of adequate response to pharmacological stress, even in patients without CAD. Previous studies have reported that the presence of SSO implies an adequate response to pharmacological stress during PET-MPI [1, 6–8]. Consistent with previous findings, our findings reinforce the potential of SSO as an important marker for evaluating the adequacy of pharmacological stress.

In the present study, we excluded patients with CAD, allowing us to conclude that reduced MFR can be attributed to either CMD or an inadequate pharmacological stress response. Among the 63 patients analyzed, 27 showed MFR of <2.0 (Fig. 3). Notably, in the negative SSO group, 80.0% of the patients showed a reduced global MFR, suggesting that this group includes not only patients with CMD but also patients with inadequate pharmacological stress. These results suggest that the absence of SSO may be useful in differentiating non-responders to pharmacological stress.

In Fig. 3, group B, with positive SSO and normal MFR, may reasonably be classified as the “normal” group. Group D, with positive SSO and reduced MFR, demonstrated an adequate response to pharmacological stress, as indicated by positive SSO [1, 6–8]. This suggests that CMD may be the cause of the reduced MFR. There were only three cases in group A, with negative SSO and normal MFR, while the mean global MFR was 2.54. A global MFR of ≥2.0 suggests an appropriate response to pharmacological stress; however, because SSO is negative, maximum hyperemia may not have been achieved, and the actual global MFR may be higher than the recorded value. In group C, which had negative SSO and reduced MFR, both CMD and an inappropriate response to pharmacological stress may have been involved. By incorporating SSO into clinical protocols, clinicians can more accurately distinguish CMD as the cause of reduced MFR in patients without CAD, thereby refining the diagnostic specificity for CMD and enhancing the interpretation of MFR.

In addition, assessing the adequacy of response to pharmacological stress is crucial for the precise diagnosis of ischemic heart disease. Traditionally, increased HR and decreased BP have been considered reliable markers of adequate pharmacological stress [21]. However, these hemodynamic parameters alone may not always accurately reflect pharmacological stress responses. BP is an inaccurate marker for ATP and adenosine stress tests. This is because these tests require lower limb BP measurements. In practice, in the present study, BP was measured at the ankle, and in four patients, it was impossible to measure. Typically, both upper limbs are used as routes for the continuous intravenous injection of short-half-life agents, such as ATP or adenosine, and for the injection of radiotracers. However, we also consider HR changes and BP changes to be important markers of an appropriate response to pharmacological stress. In the present study, when we investigated the relationship with SSO, there was no significant difference, but this may have been affected by the small sample size. If the sample size is larger, the combination of SSO, HR change, and BP change may become a more powerful marker of appropriate pharmacological stress.

Patriki et al. demonstrated that the absence of SSO was not a reliable indicator of adenosine-induced coronary vasodilation failure [7]. We agree that a negative SSO does not necessarily lead to the conclusion that the patient failed to respond to pharmacological stress. However, in the present study, we excluded patients with CAD; therefore, if the SSO was negative, we should at least consider the possibility of an inadequate response to pharmacological stress as a cause of reduced MFR. On the contrary, if SSO is positive, the response to pharmacological stress is adequate, as in previous studies [1, 6–8]. The presence or absence of SSO may be a useful reference for evaluating MBF and MFR.

In our study, we also evaluated quantitative SSO using SRR. Using previous report as a method of quantification, we used a cutoff of 0.88 for the SRR as an indicator of responder [1]. However, it is difficult to determine the same cutoff value across different PET modalities, tracers, and pharmacological stress agents. A previous study showed no significant differences between visual and quantitative assessments of SSO in terms of evaluation via MFR and identification of non-responders to pharmacological stress [1]. Ammonia PET is available in a variety of method, with the advantage that visual evaluation is simple and can be measured at any facility. In clinical practice, visual SSO can be easily identified when writing a reading report. We believe that visual assessment is sufficient for evaluating SSO.

Chest pain can occur even in the absence of CAD, a phenomenon increasingly identified as INOCA [20, 22, 23]. One underlying cause is CMD. CMD, characterized by impaired microvascular function, can lead to a reduced MFR even in the absence of significant epicardial coronary artery stenosis [24–26]. Thus, even in the absence of an ischemic pattern (fill-in) in the qualitative polar map of stress and rest PET perfusion imaging, a reduced MFR may indicate CMD. Conversely, a positive fill-in indicates CMD regardless of the MFR value. CMD is associated with increased mortality and MACE [27–29]. When both ischemic patterns (fill-in) and reduced MFR are present, the risk of MACE increases even further [22].

SSO may also be useful for INOCA risk assessment, and Fig. 6 shows the flowchart of the clinical decision pathway for INOCA using PET-MPI, including SSO [20]. In cases of suspected INOCA, a reduced MFR should not be easily evaluated as an increased risk of MACE. In our clinical decision pathway, it is necessary to incorporate the presence or absence of SSO into the evaluation, considering the possibility of insufficient pharmacological stress.

By leveraging SSO in conjunction with other clinical and imaging parameters, clinicians can achieve more comprehensive risk stratification and tailor management strategies more effectively for patients without CAD. This approach enhances the precision of MFR assessment and aids in identifying patients who may benefit from targeted therapeutic interventions aimed at improving microvascular function. Future studies should continue to explore the integration of SSO with advanced imaging modalities to further refine the diagnostic workflow and optimize patient outcomes in this population.

## Limitations

This study has some limitations. First, the relatively small sample size may have limited the generalizability of our findings. Larger studies with more extensive cohorts are required to validate our results. Second, the single-center design may not be representative of broader populations because patient demographics and clinical practices can significantly vary across different institutions. Therefore, multicenter studies are required to confirm the applicability of SSO in various clinical settings. Further studies should explore standardized protocols to minimize this variability and enhance the reliability of SSO in clinical practice.

Additionally, there were significant differences in baseline characteristics, such as sex, smoking, and CKD, which may have influenced the presence of SSO. However, no previous studies have reported on the effects of these variables on SSO. This limitation may be attributed to the small sample size in this study.

Third, in the present study, ICA was performed in some cases before confirming ischemia via PET-MPI, and coronary flow reserve (CFR) and index of microcirculatory resistance (IMR) measurements and provocation testing were not conducted in all cases. However, the strict diagnosis of INOCA necessitates comprehensive assessment, including measuring CFR, IMR, and vasoreactivity provocation during ICA [23]. Future investigations focusing on patients definitively diagnosed with INOCA are warranted to address these limitations and further elucidate optimal diagnostic and management strategies.

Finally, our institution did not analyze cardiac function using ECG gating during the ^13^N-ammonia PET scan. The relationship between the SSO and ejection fraction would also be an issue for future studies.

## Conclusions

SSO positivity is associated with higher global stress, MBF, and MFR in patients without CAD, suggesting its potential utility as a marker for adequate pharmacological stress. Furthermore, CMD has been suggested as a potential cause of reduced MFR in patients without CAD. These findings support the use of the SSO in conjunction with other clinical and imaging parameters to improve the accuracy of MFR assessments and risk stratification in patients without CAD.

## Supplementary Information

Below is the link to the electronic supplementary material. The ppt file contained memo note. Please delete them when uploading or convert to PDF..

Supplementary file1 (DOCX 24 KB)

Supplementary file 2 (PPTX 154 KB)
