# Supplementary material for: Clinical significance of splenic switch-off in adenosine triphosphate 13N-ammonia positron emission tomography in patients without coronary artery disease
Source: Jpn J Radiol. 2025 Mar 29;43(7):1186–96. doi: 10.1007/s11604-025-01762-0 (PMC12204888; doi:10.1007/s11604-025-01762-0)
Supplement: Supplementary file 1 — Supplementary file1 (DOCX 24 KB) [file 11604_2025_1762_MOESM1_ESM.docx]

**Supplemental data**

**Supplementary Table 1. Global MFR stratified by SSO and increased HR**

|  | increased HR (≥ 10) | unchanged HR (<10) | P-value |
| --- | --- | --- | --- |
| Positive SSO | 2.3 ± 0.6 | 2.6 ± 0.7 | 0.26 |
| Negative SSO | 1.7 ± 0.7 | 1.5 ± 0.5 | 0.57 |

Data given as mean ± SD

*MFR* myocardial flow reserve, *SSO* splenic switch-off, *HR* heart rate

**Supplementary Table 2. RPP-corrected global rest MBF and MFR**

|  | Negative SSO  (n=13) | Positive SSO  (n=47) | P-value |
| --- | --- | --- | --- |
| RPP (mmHg·bpm) | 9812.7 ± 2888.9 | 9961.5 ± 2352.9 | 0.85 |
| corrected global rest MBF  (ml·min^-1^·g^-1^) | 1.1 ± 0.4 | 1.0 ± 0.3 | 0.75 |
| corrected global MFR | 1.5 ± 0.4 | 2.3 ± 0.6 | < 0.001 |

Two patients from the group without SSO and one patient from the group with SSO were excluded from the analysis due to missing systolic blood pressure.

Data given as mean ± SD

*RPP* rate pressure product, *bpm* beat per minute, *MBF* myocardial blood flow, *MFR* myocardial flow reserve
