# Supplementary material for: Clinical significance of splenic switch-off in adenosine triphosphate 13N-ammonia positron emission tomography in patients without coronary artery disease
Source: Jpn J Radiol. 2025 Mar 29;43(7):1186–96. doi: 10.1007/s11604-025-01762-0 (PMC12204888; doi:10.1007/s11604-025-01762-0)
Supplement: Supplementary file 2 — (PPTX 154 KB) [file 11604_2025_1762_MOESM2_ESM.pptx]

## Slide 1
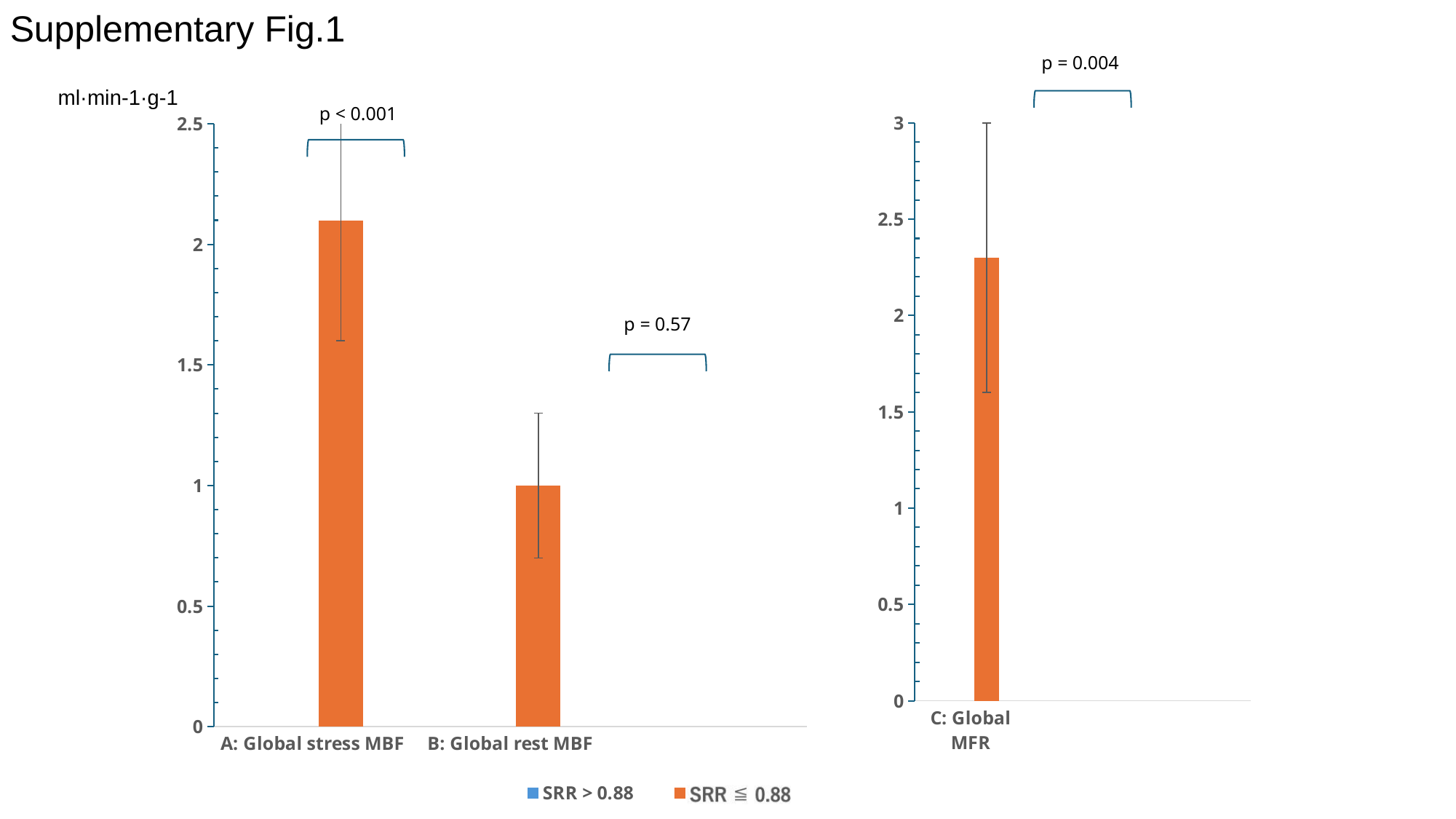

Supplementary Fig.1
p = 0.004
ml·min-1·g-1
### Chart
| Category | SRR > 0.88 | SRR ≦ 0.88 |
|---|---|---|
| A: Global stress MBF | 1.5 | 2.1 |
| B: Global rest MBF | 1.0 | 1.0 |
### Chart
| Category | negative SSO | positive SSO |
|---|---|---|
| C: Global MFR | 1.6 | 2.3 |p < 0.001
p = 0.57

## Slide 2
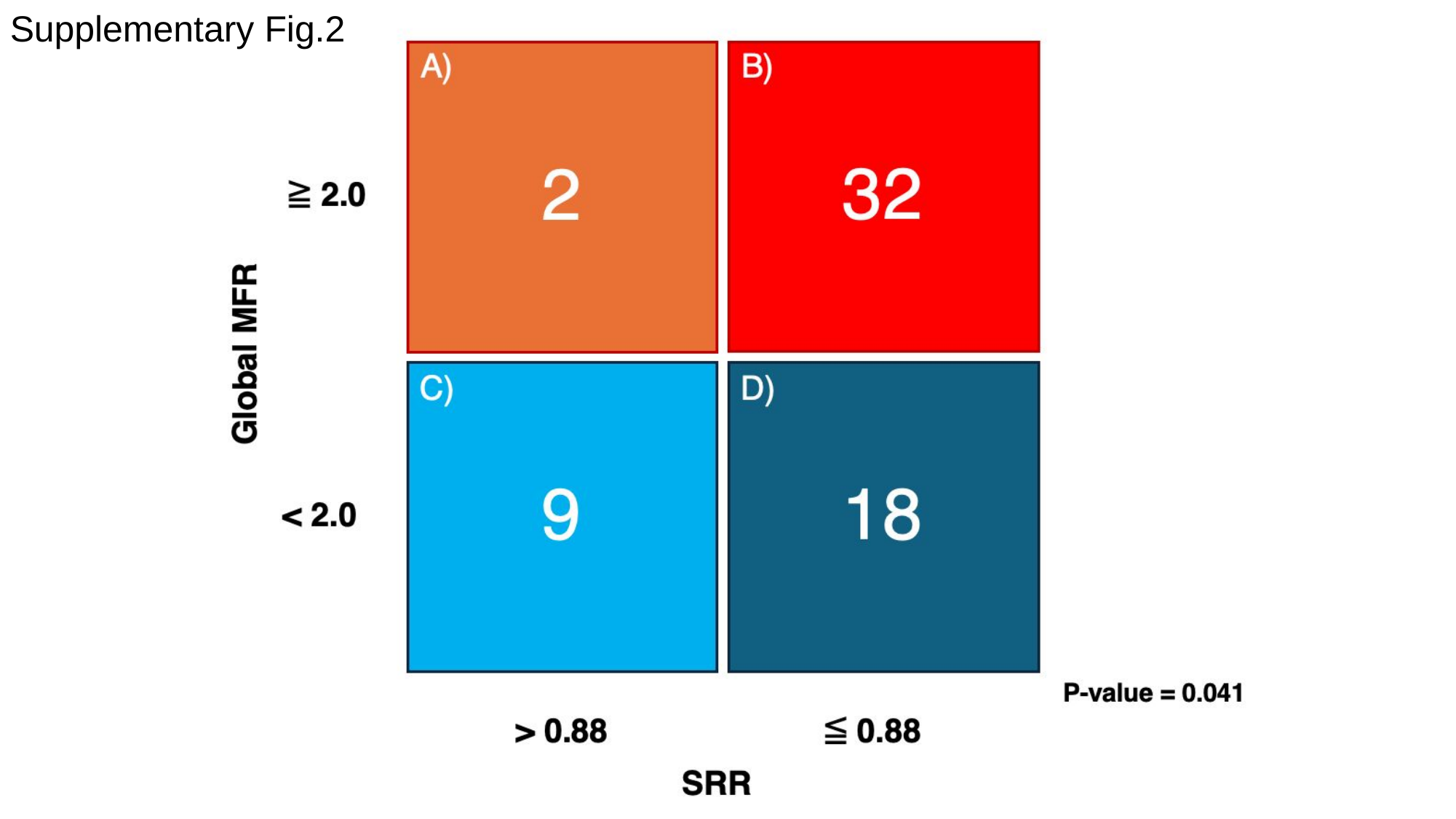

Supplementary Fig.2
